# Methods for Determining the Number of Mold Fungi in the Air—Current Trends

**DOI:** 10.1111/1758-2229.70386

**Published:** 2026-07-05

**Authors:** Juhi Mishra, Wioletta Przystas

**Affiliations:** ^1^ Department of Air Protection, Faculty of Energy and Environmental Engineering Silesian University of Technology Gliwice Poland

**Keywords:** air sampling methods, fungal bioaerosol, indoor air

## Abstract

Indoor fungal contamination poses significant health concerns due to the diversity and dynamic behaviour of airborne spores. Accurate quantification of indoor fungal bioaerosols remains challenging because of variability in sampling efficiency, particle‐size resolution, and analytical approaches. This review critically evaluates commonly used physical sampling methods, including Andersen cascade impactors, Coriolis μ liquid cyclones, sedimentation techniques, and filter‐based air sampling, with emphasis on volumetric accuracy, reproducibility, and compatibility with downstream molecular analysis. The processes underlying spore aerosolization in indoor environments and their implications for air quality and occupant exposure are discussed. Indoor fungi such as Aspergillus, Penicillium, Cladosporium, and Rhizopus proliferate under high humidity and inadequate ventilation, particularly within damp materials and HVAC systems. Despite technological advances, significant gaps persist in methodological standardization, harmonization of exposure metrics, and integration of indoor–outdoor fungal dynamics. Such heterogeneity limits cross‐study comparability and constrains exposure assessment. By systematically comparing available methods and their optimal applications, this review highlights the need for integrated and standardized strategies to improve fungal bioaerosol quantification and support evidence‐based indoor air quality management.

## Graphical abstract

A unified methodological framework combining culture‐based, molecular, and data‐driven approaches to advance reliable measurement and interpretation of indoor fungal bioaerosols.

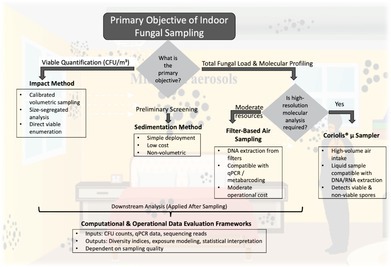

## 1 Introduction

Air pollution remains a critical global environmental issue affecting both outdoor and indoor air quality. While research has traditionally focused on chemical pollutants and particulate matter, increasing attention has been directed toward biological contaminants. Indoor air quality is shaped by complex interactions among outdoor air infiltration, building materials, ventilation characteristics, occupant behaviour, and microbial colonization, especially in modern buildings that are increasingly airtight and densely occupied.

Microorganisms present in indoor air, and their metabolic by‐products, are now widely recognized as important contributors to indoor air pollution. Apart from pathogenic bacteria, we should also remember about mould fungi and their spores. While low concentrations of non‐pathogenic microorganisms are generally considered harmless, elevated microbial levels can compromise indoor environmental safety. Such conditions are especially concerning when microbial loads exceed established hygienic thresholds, as they may contribute to respiratory disorders, allergic reactions, and other adverse health effects. Vulnerable populations, including children, older adults, and immunocompromised individuals, are particularly susceptible to these impacts (Kumar et al. 2021).

This concern is remarkably pronounced in high‐occupancy public buildings such as schools, universities, daycare centres, hospitals, and sports complexes, where occupants, especially children, are continuously exposed to different airborne microorganisms (Hänninen 2011; Kumar et al. 2021). Due to their lower body weight and higher breathing rates, children inhale a greater volume of air per kilogram of body mass than adults, resulting in increased exposure to airborne contaminants. In addition, their developing immune and respiratory systems make them more vulnerable to the adverse health effects associated with indoor bioaerosols (Hänninen 2011). Therefore, maintaining microbial safety in indoor environments is not only a matter of environmental hygiene but also a critical component of public health protection and healthy development (Hänninen 2011).

Although numerous studies have documented the occurrence of indoor fungal contamination and associated health concerns, there remains a lack of clear methodological guidance on how airborne indoor fungi should be quantitatively assessed. Existing literature often reports fungal presence without systematically comparing sampling approaches in terms of volumetric accuracy, usability, cost, and suitability for different exposure scenarios. This review addresses this gap by critically evaluating commonly used indoor air sampling methods for fungi, comparing their practical performance, limitations, and application contexts, and providing a structured comparison to support informed method selection for indoor fungal assessment (Li and Yang 2004).

### 1.1 The Most Important Health Effects of Indoor Fungi

Exposure to fungal spores has long been associated with the onset and exacerbation of atopic asthma, with fungal sensitization contributing to a spectrum of clinical outcomes ranging from mild allergic reactions to severe asthma. Allergic Bronchopulmonary Mycosis (ABPM) represents a well‐defined asthma endotype driven by fungal allergy. Several other fungal‐associated allergic and asthma‐like health problems have also been described, including Severe Asthma with Fungal Sensitisation (SAFS), Allergic Fungal Airways Disease (AFAD), airway mycosis, and fungal asthma. Additional fungal‐related health issues include thunderstorm asthma and Allergic Fungal Rhinosinusitis (AFRS). Thunderstorm asthma refers to acute asthma exacerbations triggered by the combined effects of airborne fungal spores and meteorological conditions during thunderstorms, whereas AFRS is a predominantly nasal allergic disorder that frequently co‐occurs with allergic rhinitis and asthma (Hadebe and Brombacher 2019).

Of special concern are opportunistic fungal pathogenic infections, which are capable of causing invasive infections in immune compromised hosts (Latgé and Chamilos 2019). In such cases, inhaled spores can bypass the immune defence and establish infections in the lungs or other tissues, resulting in conditions such as aspergillosis. The growing evidence linking indoor mould exposure to both acute and chronic health outcomes highlights the urgent need for systematic monitoring and control strategies aimed at improving indoor air quality in both residential and public buildings (Tran et al. 2020).

Globally, the prevalence of fungal sensitization varies widely, affecting ~3%–10% of the general population, 7%–20% of individuals with asthma, 35%–75% of patients with severe asthma, and up to 54%–91% of those with life‐threatening asthma. Collectively, this evidence highlights the need for continued research to identify clinically relevant allergenic fungal species and to develop targeted therapeutic strategies for managing fungal‐induced atopic disease (Agarwal and Gupta 2011; Wardlaw et al. 2021).

### 1.2 Fungi That Dominate in Outdoor Environment

Where you live and what time of year it is plays a big role in what fungal spores you're breathing in both inside your home and outside. Outdoor spores tend to be far more abundant than indoor ones, especially in dry interior spaces. Scientists have spent years studying how outdoor spore levels shift across seasons in different parts of the world, and what they've found is that it's never just one thing driving these changes (Garrett et al. 1998). Weather patterns, the types of plants growing nearby, and human activities like farming and large‐scale composting all feed into how fungi grow and release their spores, and the way these factors interact is still not completely understood, even for the species we know cause allergies. Many allergy‐triggering fungi are tied closely to agricultural settings. *Alternaria*, for example, tends to show up around potato and tomato crops, while *Cladosporium* is linked to cereal plants, and *Aspergillus* thrives in decaying plant matter and compost heaps (Bardin and Gullino 2020). For other fungal groups, the seasonal story is less clear, though we do have a better picture for basidiomycetes, the kind of fungi that produce visible fruiting bodies, which makes it easier to track when they're sporulating. One interesting pattern that's emerged from indoor‐outdoor comparisons is the ratio of *Aspergillus*/*Penicillium* to *Cladosporium* spores roughly 3:2 indoors and flipped to 2:3 outdoors, suggesting that which fungus dominates really does depend on where you're measuring (Anees‐Hill et al. 2022; Haas et al. 2014; Qi et al. 2020; Minahan et al. 2022).

Peak spore counts indoors were recorded in January, while outdoors the numbers peaked in October, and in both cases *Cladosporium* was largely responsible for those spikes. This isn't surprising; *Cladosporium* keeps showing up as the dominant airborne fungus across a wide range of environments. Studies in French sawmills processing softwood, Polish sawmills, and outdoor air surveys across India and other tropical countries have all pointed to the same genus sitting at the top of the list (Jothish and Nayar 2004; Haas et al. 2014). Other spore types, including basidiospores, ascospores, and those from genera like *Ganoderma* and *Nigrospora*, were found at concentrations at least twice as high outdoors as indoors. The likely reason is simple: indoors, there just isn't enough of the right material for these fungi to grow on, so they're not really living there. When you do find them inside, they've almost certainly drifted in through an open window or door. *Ganoderma* and other basidiospores were especially prevalent during the rainy season, typically between July and November, a pattern that makes sense given how much moisture drives fungal activity (Nayar and Jothish 2013).

### 1.3 Fungi That Dominate in Indoor Environment

Indoor environments with limited air exchange promote the accumulation of moisture and bioaerosols, thereby creating conditions favourable for fungal growth. Damp indoor environments commonly support the proliferation of fungal genera such as *Aspergillus*, *Cladosporium*, *Penicillium, Stachybotrys, Alternaria, Epicoccum*. Spores released from these environments can exacerbate many respiratory conditions, like asthma and rhinitis, with pathogenic species such as *Aspergillus fumigatus* posing particular health risks (Latgé and Chamilos 2019). Elevated concentrations of spores from fungi such as *Cladosporium* and *Serpula lacrymans* have also been associated with extrinsic allergic alveolitis. Large‐scale epidemiological studies conducted in North America and Europe demonstrate a strong association between indoor dampness or mould presence and increased respiratory symptoms. In particular, children attending preschools in damp environments have been reported to experience approximately a twofold increase in respiratory problems compared with children exposed to parental smoking. Collectively, these findings indicate that mould growth in residential settings represents a significant respiratory health concern and underscore the need for further microbiological investigations to substantiate and extend existing epidemiological evidence (Latgé and Chamilos 2019; Li et al. 2016).

**FIGURE 1:**
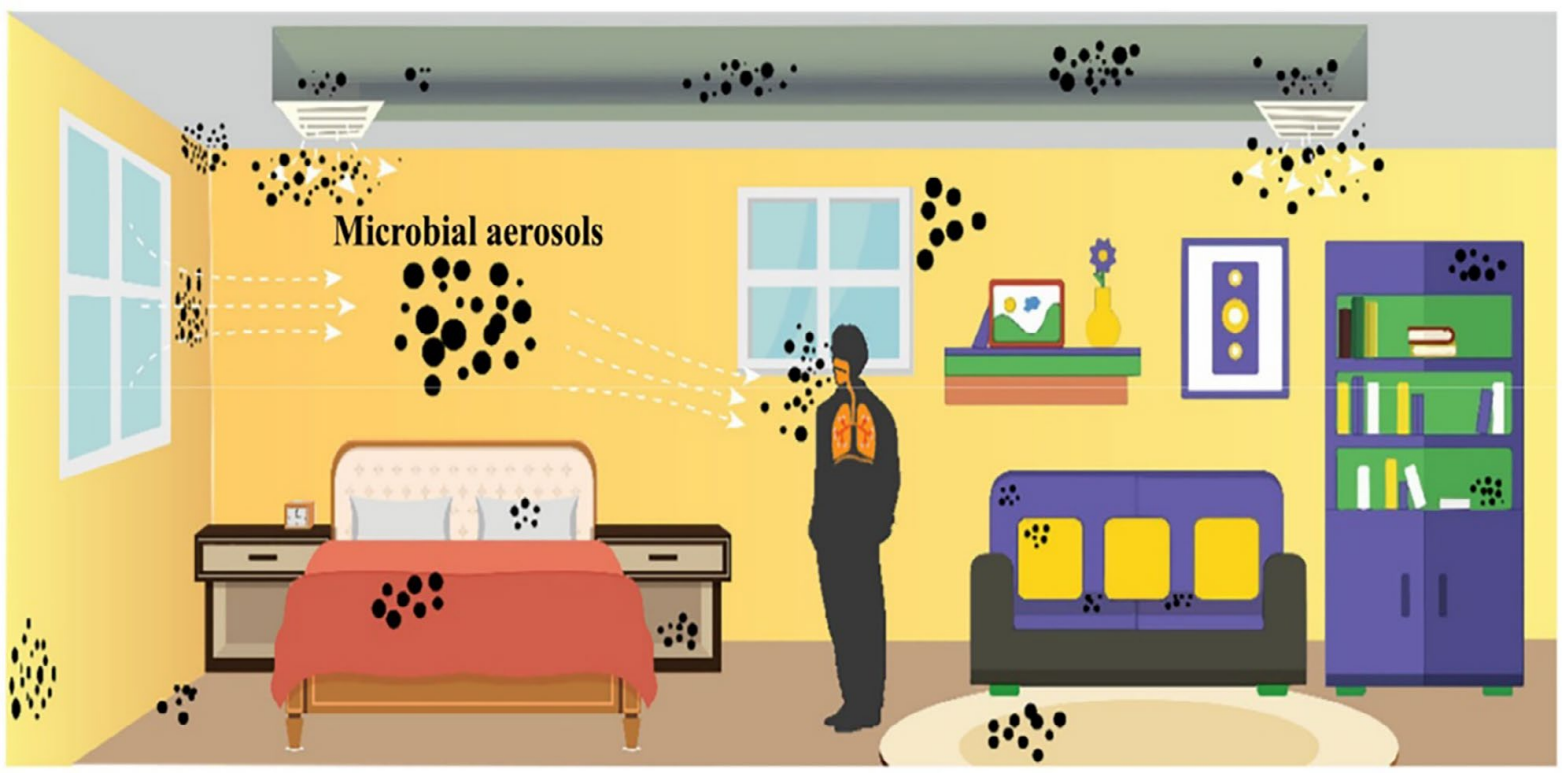
Sources of fungal bioaerosols in indoor environment and have potential for environment surface contamination (Li et al. 2022).

Sources of indoor fungal contamination (Figure 1) may include humans, pets, indoor plants, and plumbing systems. Activities and systems such as HVAC, as well as routine practices including vacuuming and mopping, can contribute to the dispersal of airborne pathogens. In addition, the resuspension of settled dust and the infiltration of outdoor air further increase the risk of microbial contamination within indoor environments (Li et al. 2022).

Airborne fungal contamination in educational environments, particularly in naturally ventilated schools, has been widely documented. Studies consistently report the dominance of common indoor genera that originate primarily from outdoor reservoirs such as soil, vegetation, and decaying organic matter and enter buildings through ventilation pathways and human activity (Małecka‐Adamowicz et al. 2020; Li and Yang 2004). Once indoors, spores may persist on surfaces including furniture, ventilation ducts, and building materials, especially where cleaning and environmental controls are inadequate. Indoor air quality in schools is shaped by ventilation type, building characteristics, occupancy density, and maintenance practices, with poorly maintained HVAC systems and overcrowding contributing to elevated microbial loads (Singha and Singha 2024; Kubba 2010). Increased fungal concentrations have been associated with allergic symptoms, respiratory irritation, and absenteeism among students and staff, highlighting the importance of routine monitoring and environmental management (Mahmoud and Jung 2025).

In addition to common classroom‐associated fungi, libraries may harbour cellulolytic species capable of degrading paper, adhesives, and archival materials (Kakde 2015). Relative humidity plays a critical role in fungal proliferation; even seasonal variations further influence airborne spore concentrations, and modest increases can promote mould growth in poorly regulated indoor conditions (Koul and Upadhyay 2018). These dynamics underscore the need for climate‐responsive ventilation, humidity control, and systematic fungal monitoring to protect both occupant health and material preservation (Hassan et al. 2021; Kakde 2015). Quantitative fungal bioaerosol measurement should therefore be viewed as a diagnostic and monitoring tool that supports risk assessment and environmental management, rather than as a standalone regulatory compliance metric.

### 1.4 Industrial Applications of Fungi Monitoring

Mould monitoring in manufacturing environments, especially in industries like pharmaceuticals and food processing, is essential for maintaining hygiene and compliance with safety standards. Contamination by mould spores can compromise product quality, lead to recalls, and cause financial losses. Mould fungi affect stored crops, such as grains and fruits, by causing spoilage and producing mycotoxins. Air monitoring in storage facilities is crucial to detect and mitigate mould growth. Industries that rely on cleanroom environments, such as electronics and biotechnology, require stringent mould control to prevent contamination of sensitive processes (Fleurat‐Lessard 2017).

Despite significant advancements in controlling microbiological spoilage in beverages, microbial contaminants can still cause defects that are noticeable to consumers, leading to dissatisfaction, complaints, health impact or product rejection. The economic and environmental consequences of such spoilage incidents can be substantial (Sohlberg et al. 2022). Modern breweries, which produce a diverse range of beverages, face an increased risk of microbial contamination from multiple sources. Microorganisms, particularly fungi, tend to colonize areas with moisture and nutrients that are challenging to clean and disinfect. Studies estimate that 95% of yeast‐related spoilage incidents in soft drinks result from poor factory hygiene (Riedl et al. 2019). In breweries and soft drink factories that do not use in‐pack pasteurization, spoilage issues often originate from filling machines and surrounding environments that support microbial growth and biofilm formation. Fungi have been detected in brewery biofilms and are among the first microorganisms to adhere to filling line surfaces after cleaning. These pioneer organisms facilitate the attachment of other microbes, including spoilage species, even though they may not necessarily grow in beer themselves (Sohlberg et al. 2022).

Sawmills and woodworking facilities represent important occupational environments for studying airborne fungal bioaerosols as well. The processing and storage of wood materials generate substantial organic dust and create favourable conditions for fungal growth, particularly under conditions of high humidity and limited ventilation. Studies conducted in sawmills across different geographic regions consistently report diverse airborne fungal communities frequently dominating indoor air samples. Seasonal variability has also been observed, reflecting changes in environmental conditions and substrate availability. Indoor concentrations in sawmills are often reported to exceed outdoor levels, likely due to the continuous presence of wood, sawdust, and temporarily moist surfaces that support fungal colonization. Mechanical disturbance and airflow within enclosed workspaces facilitate spore release and dispersion. However, reported concentrations and dominant taxa vary depending on environmental factors, climatic conditions, and the sampling methods employed. These findings highlight the importance of selecting appropriate volumetric and time‐resolved sampling strategies when assessing occupational exposure in wood‐processing environments (Dias et al. 2022; Straumfors et al. 2025).

## 2 Relevance of Quantification for Health and Safety Standards

The most significant patterns observed in relation to health include extended fungal season durations and reduced peak concentrations of fungi in warmer regions of Europe and with increasing temperatures. These findings suggest that as global temperatures continue to rise, fungal seasons in northern Europe may increasingly resemble those of southern Europe (Grinn‐Gofroń et al. 2019). Prolonged fungal seasons lead to extended exposure periods, potentially increasing the number of days individuals experience allergy symptoms. However, reductions in spore concentrations are only beneficial when levels fall below clinically relevant thresholds. In the majority of studied locations, peak concentrations of *some fungal* spores remained above clinical thresholds, even in the warmer southern European regions (Mbareche et al. 2019; Walser et al. 2015).

Accurate quantification of fungal spore concentrations is essential for establishing clinically relevant thresholds and informing health and safety standards. Long‐term monitoring (spanning decades) is necessary to provide comprehensive daily spore concentration data across a wider range of fungal taxa (Smith et al. 2023). This information is crucial for assessing the impact of climate change on fungal spore seasonality and associated changes in exposure levels that may influence public health. Given that the health effects of fungal spores are often species‐specific, further research is needed to determine threshold levels for different taxa. Notably, the only long‐term study on fungal spores identified a positive correlation between total and peak concentrations and rising temperatures, highlighting the potential for climate‐driven shifts in exposure risks (Almaguer et al. 2021).

### 2.1 Regulatory Compliance

Ensuring regulatory compliance for fungal bioaerosols is critical for protecting public health, particularly in both outdoor and indoor environments. Despite the well‐documented health risks associated with fungal spores, there are currently no universally accepted regulatory thresholds for airborne fungal concentrations (Li and Yang 2004). Existing guidelines, such as those provided by the World Health Organization (WHO) and various national environmental health agencies, primarily focus on general air quality parameters but lack standardized limits for specific fungal taxa. The World Health Organization (WHO) 2021 Air Quality Guidelines for particulate matter (PM2.5 and PM10) establish globally recognized health‐based reference values, recommending that PM2.5 concentrations not exceed 5 μg/m^3^ annually and 15 μg/m^3^ over 24 h, while PM10 levels should remain below 15 μg/m^3^ annually and 45 μg/m^3^ over 24 h (Mehta et al. 1996). These thresholds are designed to minimize adverse health effects associated with both short and long‐term exposure to airborne particulate matter. However, the implementation, regulation, and enforcement of these standards vary significantly across different geographic regions due to disparities in economic development, regulatory frameworks, infrastructure, and environmental conditions (Europe 2010).

In the European Union (EU), for instance, current air quality standards remain less stringent than WHO recommendations, with PM2.5 limited to 25 μg/m^3^ annually and PM10 to 40 μg/m^3^, though ongoing legislative revisions reflect efforts to progressively align with WHO targets. The EU acknowledges the public health burden posed by fine particulate pollution and has initiated clean air programs and regional monitoring networks to mitigate exposure (Europe 2010).

Problems related to the standards for the number of microorganisms in indoor air concern, for example, Poland. Because indoor air quality cannot be worse than outdoor air, atmospheric air values are often adopted from withdrawn standards, such as PN‐89/Z‐04111/02 Air purity protection (Łuszczyńska 2022). Microbiological tests for the determination of the number of bacteria in atmospheric air (immission) during sampling using the aspiration and sedimentation methods, and PN‐89/Z‐04111/03 air purity protection. Both of these standards were withdrawn without replacement on August 13, 2015. In the case of these standards, air is considered unpolluted if the number of mesophilic bacteria is lower than 1000 cfu/m^3^ and fungi if it is lower than 3000 cfu/m^3^. For measurements in cleanrooms, PN‐EN ISO 14698‐1:2004 Cleanrooms and associated controlled environments. Biocontamination control. Part 1. Main principles and methods applies, but it only covers the measurement principles and methodological basis for biocontamination control in cleanrooms, for example, in hospital operating rooms, without taking into account limit values (Górny 2004, 2010).

Therefore, in the case of rooms, assessments are often made based on the Krzysztofik guidelines, which consider, for example, school rooms depending on their purpose (Krzysztofik 1992). For example, in a lecture hall, the total number of bacteria on MPA medium should not exceed 1500, and the number of moulds on Sabouraud medium should not exceed 200 cfu/m^3^. For gymnasiums, these limits are 3000 and 300 cfu/m^3^, respectively. In the case of residential buildings, these limits are 1000 and 100 cfu/m^3^, respectively, for bedrooms. However, in 2004, the Team of Experts on Biological Factors of the Inter‐Ministerial Commission for Maximum Permissible Concentrations (MPC) and Maximum Permissible Intensities (MPI) of Factors Harmful to Health in the Workplace proposed much higher permissible values: 5000 cfu/m^3^ each for residential and public spaces (Chmiel et al. 2015; Górny 2010).

In the United States, the Environmental Protection Agency (EPA) sets the National Ambient Air Quality Standards (NAAQS) for PM2.5 at 12 μg/m^3^ (annual) and 35 μg/m^3^ (24‐h), while the 24‐h limit for PM10 is 150 μg/m^3^, with no annual standard for PM10. Although these values are more protective than those in some developing nations, they still exceed WHO guidelines, particularly in areas with dense urbanization and industrial activity (US EPA 2020). The WHO Guidelines for Indoor Air Quality, dampness and mould do not establish quantitative spore limits. Instead, they emphasize prevention of dampness, adequate ventilation, and prompt remediation of visible mould growth as primary interventions, regardless of measured fungal concentration for minimizing health risks, highlighting that the presence of visible mould or dampness warrants action regardless of measured spore concentrations (Heseltine et al. 2009).

In contrast, regions in South and Southeast Asia, including India, Bangladesh, Pakistan, and Indonesia, frequently experience ambient and indoor PM concentrations that far exceed both WHO guidelines and their own national standards. In major Indian metropolitan areas, for example, average PM2.5 concentrations often range between 40 and 100 μg/m^3^, driven by vehicular emissions, biomass combustion, industrial pollutants, and inadequate infrastructure. These high particulate burdens, combined with persistent indoor dampness and poor ventilation, significantly increase the risk of respiratory illness, especially where fungal contamination is present. The binding of fungal spores and hyphal fragments to PM2.5 and PM10 particles further exacerbates health risks, particularly in sensitive populations such as children, the elderly, and immunocompromised individuals (Sharma et al. 2024).

In Africa and Latin America, limited air quality monitoring capacity and widespread reliance on solid fuels for cooking and heating contribute to elevated indoor PM concentrations. In informal urban settlements and rural homes, substandard building materials and insufficient ventilation promote conditions conducive to both high particulate exposure and mould growth. Countries such as Brazil, Kenya, and Nigeria are beginning to develop air quality management frameworks, yet enforcement and data availability remain insufficient for comprehensive risk mitigation (Fisher et al. 2021). By contrast, high‐income nations such as those in Scandinavia, Japan, and Canada typically report PM levels closer to WHO recommended values, supported by stringent environmental regulations, high building standards, and widespread use of clean energy sources. These values represent proposed environmental or clinical reference thresholds reported in the literature and should not be interpreted as globally enforceable regulatory standards (Jones 2023; Hänninen 2011).

For indoor environments, regulatory compliance is particularly important in occupational and residential settings where prolonged exposure to fungal bioaerosols can contribute to health problems. Guidelines for indoor air quality (IAQ) often recommend maintaining fungal spore concentrations at levels comparable to or lower than outdoor air. However, the absence of enforceable exposure limits complicates efforts to regulate indoor fungal contamination. Institutions such as the Occupational Safety and Health Administration (OSHA) and the Environmental Protection Agency (EPA) provide general IAQ recommendations, but there is a pressing need for more specific regulatory policies that account for factors such as building ventilation, moisture control, and fungal species composition (Vornanen‐Winqvist et al. 2020).

To enhance regulatory compliance, long‐term monitoring of fungal bioaerosols should be incorporated into health and safety policies, particularly in high‐risk environments such as hospitals, schools, and workplaces with high humidity levels. Standardized methodologies for fungal quantification, combined with improved climate adaptation strategies, will be essential in ensuring that both indoor and outdoor air quality standards effectively protect public health in the face of climate change and evolving environmental conditions (Walser et al. 2015).

### 2.2 Risk Mitigation and Prevention

Effective risk mitigation and prevention strategies for fungal bioaerosols are essential to safeguard public health, particularly in environments prone to elevated fungal spore concentrations. Priority should be given to moisture prevention, building envelope integrity, adequate ventilation rates, and rapid remediation of water damage, as these remain the most effective and evidence‐supported interventions. In both outdoor and indoor settings, controlling environmental factors such as temperature, humidity, and ventilation can significantly reduce fungal proliferation and airborne dissemination (Ghosh et al. 2022).

For indoor environments, prevention strategies should prioritize moisture control through proper building design, routine maintenance, and the use of dehumidifiers or air filtration systems to minimize fungal growth. High‐risk areas such as hospitals, schools, and workplaces must implement stringent IAQ management protocols, including regular inspections for water damage and microbial contamination (Hurraß et al. 2025).

In outdoor environments, integrating fungal bioaerosol data into public health policies can help mitigate risks associated with prolonged exposure, particularly for individuals with respiratory conditions such as asthma and allergic rhinitis. Public awareness campaigns and real‐time spore monitoring systems can further aid in risk reduction by informing individuals of high‐exposure periods and promoting preventive actions, such as limiting outdoor activities when fungal spore concentrations exceed clinical thresholds (Ziaei et al. 2025).

## 3 Air Sampling Methods

When it comes to collecting fungal spores from the air, researchers generally choose between two broad approaches: active and passive sampling depending on what their study is trying to find out. Whatever method is used, the collected material usually contains a mix of spores, fungal threads (hyphae), and broken fragments of fungal matter. Active sampling uses a pump to pull air through a collection device. Depending on the setup, air might pass through a filter, get bubbled through a liquid (impinger sampling), or be directed onto a solid surface (impactor sampling). These setups work well for long‐term studies because the airflow rate is known and can be adjusted, including to match the rate at which a person naturally breathes. Passive sampling, on the other hand, simply lets particles fall out of the air onto a surface under gravity. It's cheaper and easier to set up, but it comes with trade‐offs: it tends to capture larger particles more readily, and because there's no controlled airflow, it's harder to convert results into meaningful airborne concentration figures (Senanayake 2020).

Once samples have been collected, the next step is identification and here again, researchers face a choice. Culture‐based methods grow living fungi on nutrient media to see what's viable, but they come with an inherent bias problem: different fungi have very different nutritional needs, so some species will thrive in culture while others won't even show up. Non‐viable approaches, particularly microscopy, are still the most widely used; you examine and count spores directly without trying to grow them. For viable fungal quantification, the Andersen cascade impactor is generally considered the gold standard, especially when particle size matters. But when the goal is to get a broad picture of which species are present including non‐viable material, molecular techniques like filter‐based qPCR or sequencing tend to do a better job. The right method really comes down to what you're trying to achieve: counting living fungi, mapping species diversity, or getting a quick initial read on exposure levels. In allergy research, the non‐viable route still has plenty of relevance, because spores don't need to be alive to trigger an allergic reaction. That said, microscopy has real limitations; it can only reliably distinguish a handful of fungal groups, and it takes a trained eye to do it well (Fernández‐Rodríguez et al. 2014; Knoll et al. 2023).

High‐throughput sequencing has opened up new possibilities in recent years. By targeting and sequencing variable regions of fungal ribosomal RNA genes, a technique called metabarcoding, researchers can now identify a far wider range of airborne fungi than was previously practical. This has been made increasingly accessible by falling sequencing costs and the steady growth of reference DNA databases to compare results against. Something as simple as where you place your air sampler also matters more than it might seem. Fungal spore concentrations change with altitude, so the height at which sampling equipment is operated affects both how many spores are captured and which types predominate. Sampling at greater heights is generally thought to give a picture that's more representative of a wider area, rather than just the immediate surroundings (DeLeon 2023).

**FIGURE 2:**
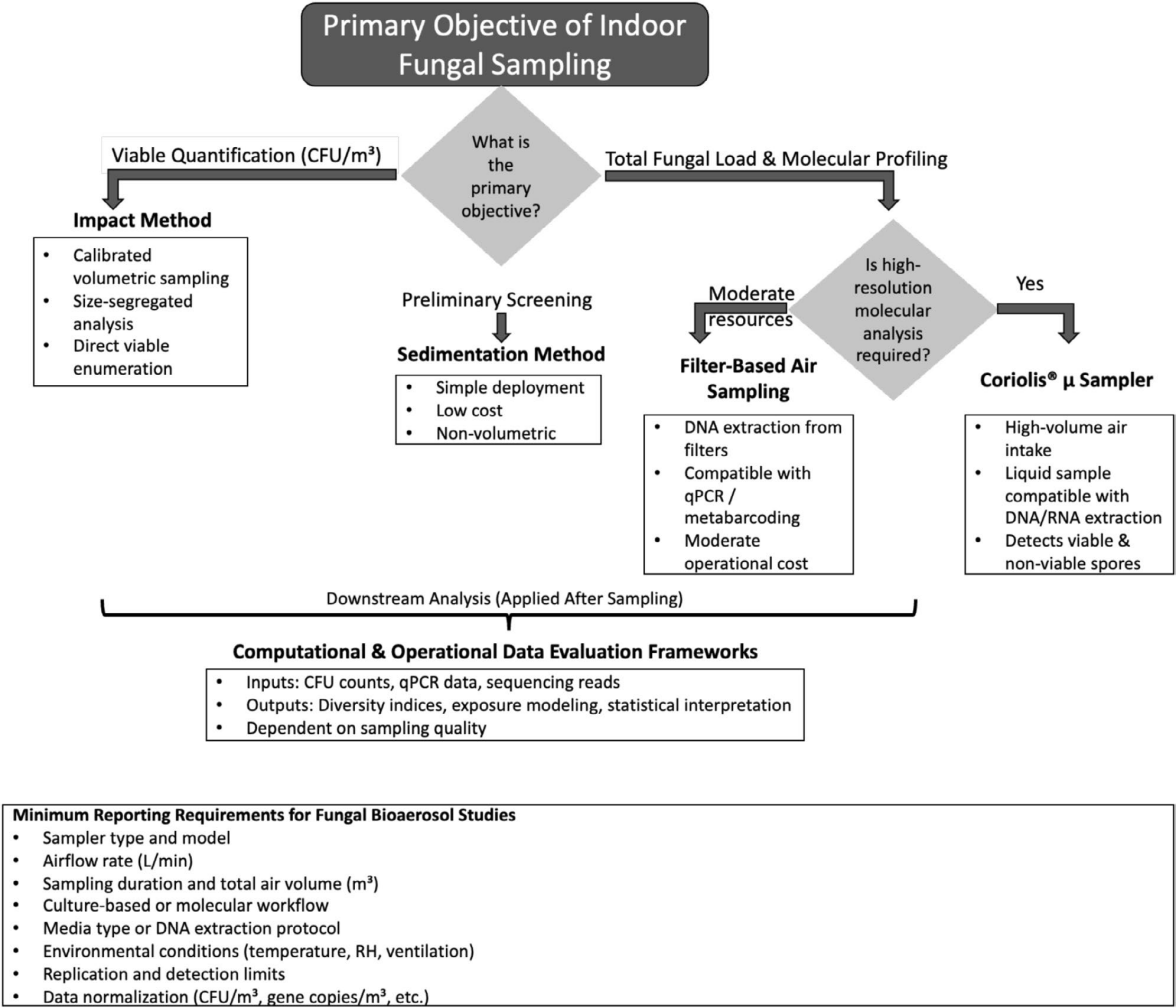
Integrated decision framework and reporting checklist that provides a structured decision tree guiding selection of sampling methods based on study objectives.

To bring all of this together in a practical way, an integrated decision framework can help researchers choose the right sampling approach from the outset (Figure 2). Structured as a decision tree, it guides users through their options based on what kind of output they need whether that's culture‐based colony counts from impactor methods, DNA‐based diversity and abundance data from filter or Coriolis μ sampling, or a simple presence/absence screen using sedimentation. Having this kind of structured guidance helps make both method selection and results interpretation more consistent, particularly in the context of indoor fungal bioaerosol assessments.

### 3.1 The Impact Method

One of the most commonly used approaches for capturing fungal bioaerosols from the air is the impaction method. The way it works is fairly straightforward: an air sampler draws in air and directs it forcefully onto a solid or semi‐solid growth medium sitting inside a collection plate. Any fungal spores or particles carried in that airstream get deposited onto the surface, where they can then be cultured and identified in the lab. What makes this method particularly useful is its precision: it can deliver quantitative data on spore concentrations per unit volume of air, and it's capable of capturing a broad spectrum of fungal types including smaller, lighter spores that other methods might miss. Once collected, the spores can be grown out in culture to identify which species are present. That said, the method isn't without its drawbacks. The physical force of impaction can damage or kill more fragile spores, which means some viable organisms may not survive long enough to grow in culture. There's also the issue of non‐viable spores: particles that are no longer alive but can still provoke environmental or health effects which this method simply cannot capture. Despite these limitations, impaction sampling remains extensively used across environmental monitoring, occupational health research, and indoor air quality assessments, where being able to accurately count and identify airborne fungi is essential for understanding exposure risks and designing appropriate controls (Vishwakarma et al. 2025).

Measuring occupational exposure to bioaerosols is not straightforward, and having reliable sampling equipment is central to getting meaningful results. A range of commercially available devices exists for this purpose, but the Andersen six‐stage and two‐stage viable particle‐sizing samplers have long been considered the benchmark tools in the field. Their main practical limitation, however, is size: they're bulky and depend on an external power source, which makes them difficult to deploy in remote or hard‐to‐reach settings. A controlled evaluation comparing eight different bioaerosol samplers inside a horizontal bioaerosol chamber found that the Andersen six‐stage, Andersen one‐stage, and Ace AGI‐30 samplers performed best when it came to recovering freely dispersed microbial aerosols (Mehta et al. 1996). In a follow‐up study conducted in an experimental room designed to identify the most effective methods for capturing fungal spores and to look at how human movement affects air sampling, the Andersen six‐stage viable impactor and the Burkard spore trap came out on top in terms of spore recovery. Building on this work, a further comparison was carried out to evaluate three samplers that had not previously been assessed against one another: the Burkard portable, the RCS Plus, and the SAS Super 90. The Andersen two‐stage impactor was used as the reference device throughout this comparison (Parker et al. 2020).

The Andersen two‐stage viable impactor pulls air through the device at a calibrated rate of 28.3 L per minute. Each of its two stages contains 200 orifices 1.5 mm in diameter on the first stage and 0.4 mm on the second. Collection efficiency in impactors like this is typically described using the Stokes number at 50% efficiency (Stk_50_), which essentially refers to the particle size at which half of all particles are expected to be captured. This is tied to the concept of the cut‐off diameter (d_50_), which assumes an even distribution of particle mass above and below that threshold. For the Andersen impactor, the d_50_ values are 8.0 μm for the first stage and 0.95 μm for the second, meaning that particles larger than these values are captured with near‐complete efficiency at their respective stages.

To ensure accurate operation, the sampler was calibrated using a rotameter that had itself been checked against a dry gas meter one that had been standardized by the Johnson Space Center Calibration Laboratory using a Brook's Bell Prover. During sampling, each stage of the impactor held a sterile petri dish filled with nutrient agar prepared to the manufacturer's specifications. Plastic tubing connected the sampler to the rotameter, and the airflow was carefully set to exactly 28.3 L per minute to keep sampling conditions consistent throughout (Grinshpun et al. 2016).

### 3.2 Coriolis μ Sampler

The Coriolis μ (Bertin Instruments, Montigny‐le‐Bretonneux, France) is a high‐performance, high‐volume cyclone air sampler designed for the collection of airborne particles into a liquid medium. Its operating principle is based on liquid scrubber aerosol collection, utilizing tangential impingement to induce a cyclonic vortex within a collector cone filled with buffer solution. As air is drawn into the cone, the generated vortex directs particles toward the inner walls by centrifugal force, thereby separating them from the airstream and concentrating them into the liquid buffer. This method facilitates efficient collection of bioaerosols and enables downstream analysis through a wide range of analytical techniques. Detailed schematics of the collection mechanism are provided in the Bertin user manual (05027–006‐DU002‐F ENG) and supporting scientific literature (Al Hallak et al. 2025).

The Coriolis μ has been increasingly adopted in microbiological air sampling due to its ability to generate liquid samples suitable for various detection methods, including culture‐based assays, nucleic acid amplification techniques (e.g., PCR, qPCR), microscopy, spoligotyping, and DNA sequencing. It has proven effective in detecting fungal spores, pollen, and a range of airborne pathogens, particularly in indoor environments and healthcare settings. Additional applications include flow cytometry, solid‐phase cytometry, and immunoassays for identifying microbes or their simulants. Notably, the Coriolis μ has also been employed in environmental forensics, such as detecting airborne traces of trinitrotoluene (TNT) via electrochemical assays. Despite its versatility, the Coriolis μ presents certain limitations. The mechanical stress associated with cyclonic sampling can result in sample loss and reaerosolization within the collector cone. These phenomena may reduce collection efficiency and compromise the integrity of the sample, potentially leading to inaccurate analytical results. Moreover, reaerosolized particles can deposit on internal surfaces of the sampler, raising the risk of cross‐contamination between samples and potential biohazard exposure to operators. These factors highlight the importance of rigorous decontamination protocols and careful handling during microbiological air sampling (Al Hallak et al. 2025; Yinon 1999).

### 3.3 The Sedimentation Method

The sedimentation method is a simple and cost‐effective technique for collecting and studying airborne fungi. It relies on the passive settling of fungal spores onto a surface, such as an open Petri dish containing a growth medium, over a specific period. This method is particularly useful for preliminary studies or in settings where resources are limited. A significant strength of the sedimentation method lies in its simplicity, affordability, and ease of implementation, as it does not require specialised equipment. It is also effective for identifying dominant fungal species in a given environment. However, its application is somewhat limited due to its inability to quantify airborne fungal concentrations accurately, as it does not account for spore deposition rates or variations in air currents. Additionally, it primarily captures larger spores that settle more readily, potentially underrepresenting smaller, lighter spores that remain airborne longer. Despite these limitations, the sedimentation method remains a valuable tool for basic fungal air quality assessments and can serve as a starting point for more advanced studies, but it is not useful for the larger area having small concentration of different spores (Datsugwai Mohammed and Balogu 2023).

### 3.4 Filter Based Air Sampling

Filter based air sampling is a widely employed active technique for the collection of airborne fungal bioaerosols in indoor, occupational, and environmental settings. In this approach, air is drawn through a porous membrane filter using a calibrated pump, allowing suspended particles including fungal spores, hyphal fragments, and associated microbial aggregates to be retained on the filter surface. The efficiency of particle capture is governed by mechanisms such as inertial impaction, interception, diffusion, and electrostatic attraction, depending on particle size and airflow conditions. A range of filter materials is available, including polycarbonate (e.g., Nuclepore), polytetrafluoroethylene (PTFE), mixed cellulose ester, cellulose acetate, glass fibre, nylon, and gelatin filters. Material selection is dictated by study objectives and downstream analytical requirements. Polycarbonate and PTFE filters are commonly used for microscopic and molecular analyses due to their defined pore size and smooth surface morphology, whereas gelatin filters are often preferred when preservation of microbial viability is critical. Glass fibre filters offer high loading capacity but may complicate DNA extraction due to matrix interference (Skjøth and Petch 2022).

Following collection, two principal analytical pathways are available. In culture based workflows, particles are eluted from the filter surface into a sterile liquid medium and subsequently plated for colony forming unit (CFU) enumeration. Alternatively, filters may be subjected directly to nucleic acid extraction for quantitative PCR (qPCR), Next Generation Sequencing (NGS), or metagenomic analyses. The latter approach enables detection of both viable and non‐viable fungal material, thereby providing a more comprehensive characterization of the indoor mycobiome compared with culture‐dependent methods alone. One of the principal advantages of filter based sampling is its versatility. The method supports both viable and total microbial assessments and can be integrated with molecular techniques to enhance taxonomic resolution. Volumetric quantification is achieved through controlled airflow rates and known sampling durations, allowing results to be expressed in cfu/m^3^ or gene copies per cubic meter. In addition, high‐volume filter samplers, including cascade systems designed for particulate matter monitoring (e.g., PM_10_, PM_2.5_, PM_1_), enable size fractionated bioaerosol analysis and facilitate alignment with regulatory particulate matter monitoring frameworks (Rastmanesh et al. 2024).

Despite these strengths, several methodological limitations must be considered. Prolonged sampling may result in desiccation stress, reducing the culturability of collected organisms and potentially underestimating viable concentrations. High particulate loading, particularly in industrial environments, can lead to filter clogging and altered flow rates, thereby affecting sampling efficiency and particle size capture. Recovery efficiency is influenced by filter material, pore size, elution technique, and storage conditions prior to analysis (Yan et al. 2025). Mechanical agitation (e.g., vortexing or sonication) is often required to optimize particle recovery from the filter matrix. Moreover, relative humidity and temperature during sampling and storage can significantly affect microbial viability and nucleic acid integrity. Filter based samplers can be broadly categorized into single filter systems and cascade configurations. Single filter devices are generally lightweight, portable, and suitable for personal exposure assessment, whereas cascade samplers permit simultaneous collection of multiple particle size fractions but require more complex post processing and higher capital investment. High volume systems are particularly advantageous when large air volumes must be sampled over extended periods or when detection of low abundance taxa, including antimicrobial resistant fungi, is required.

Recent advances in nanofibrous and electrospun filter materials have improved capture efficiency and expanded functional capabilities. Nanofiber membranes offer high surface area to volume ratios and small pore sizes, enhancing collection efficiency for submicron particles. Incorporation of antimicrobial agents, such as silver nanoparticles or metal oxide composites, has been explored to reduce post collection microbial viability and enhance biosafety; however, such modifications may also influence downstream viability based analyses and therefore require careful consideration. Filter‐based air sampling represents a robust and adaptable approach for the quantitative and qualitative assessment of airborne fungi. When appropriately selected and integrated with molecular and computational analytical methods, it serves as a critical component of comprehensive indoor bioaerosol monitoring strategies (Zhu et al. 2025).

### 3.5 Discussion and Comparison of Methods

The filter‐based sampling method, impact method, and sedimentation method for fungal bioaerosol sampling differ significantly in terms of accuracy, practicality, adaptability, and reproducibility. In terms of accuracy, the filter sampling method, which includes molecular techniques like high‐throughput sequencing (HTS) and qPCR, provides high precision by detecting a wide range of fungal species, including non‐viable spores (Mbareche et al. 2018). However, it may introduce biases due to variations in DNA extraction efficiency and database limitations. The impact method, particularly volumetric impactor sampling, is commonly regarded as a reference method for viable fungal bioaerosol quantification as it ensures reproducibility and accurate quantification by collecting airborne spores at a controlled airflow rate. This method, often analysed via microscopy or culture‐based techniques, provides reliable fungal concentration and composition data but may be limited in taxonomic resolution unless molecular techniques are applied. The sedimentation method, which relies on passive gravitational deposition, is the least accurate since it does not define air volume, leading to challenges in precise quantification. It also favours larger spores with higher deposition rates, potentially underrepresenting smaller fungal particles (King et al. 2020).

From a practicality perspective, the filter sampling method requires specialized laboratory equipment, skilled personnel, and complex bioinformatics analysis, making it less feasible for field studies or on‐site assessments despite its high accuracy. The impact method, while widely used in fungal air sampling, depends on power sources, regular calibration, and proper sample handling, which can limit its practicality in remote or resource‐limited areas. In contrast, the sedimentation method is the most practical as it requires minimal equipment, is cost‐effective, and does not need electricity or technical expertise. However, its practicality comes at the expense of accuracy and quantification reliability due to its passive nature and dependence on environmental conditions (Ngashangva et al. 2022).

In terms of adaptability, the filter‐based sampler is moderately versatile for detecting a broad range of fungal species, including non‐viable spores, but it is limited by its reliance on laboratory settings. Advances in portable sequencing technologies are improving its field applicability. The impact method is moderately adaptable, as it can be used in both indoor and outdoor environments and adjusted for different airflow rates and sampling durations. However, it requires regular maintenance and power sources, which can be a challenge in certain locations. The sedimentation method is the most adaptable in terms of deployment because it is simple, inexpensive, and does not require specialized equipment, making it suitable for preliminary assessments and long‐term passive monitoring. Nevertheless, its inability to quantify fungal concentrations accurately and its bias toward larger spores restrict its overall adaptability (Thompson et al. 1994; Haig et al. 2016).

Regarding reproducibility, the filter sampling method offers moderate consistency through standardized DNA extraction, amplification, and sequencing protocols, though variations in sample processing and database reliance can introduce discrepancies between studies. The impact method is the most reproducible for fungal bioaerosol quantification due to its controlled airflow rate and defined sampling parameters, making it ideal for comparative studies and long‐term monitoring. When combined with standardized microscopic or culture‐based analysis, it ensures reliable and repeatable results. Conversely, the sedimentation method has the lowest reproducibility as it depends on passive deposition, which can be influenced by external factors such as airflow, humidity, and turbulence. Since the volume of sampled air is unknown, direct comparisons across studies are difficult, further limiting its reliability. Overall, while the filter sampling method excels in species identification and precision, the impact method balances accuracy and field applicability, and the sedimentation method remains the most practical and adaptable but with limitations in accuracy, reproducibility, and quantification (Hardwick et al. 2017).

No statistically significant differences were observed among the samplers in their ability to recover different fungal species. The Burkard impactor collected slightly higher total fungal concentrations (expressed as colony‐forming units per cubic meter, cfu/m^3^) than the Andersen sampler across all sampling locations, with the exception of the electrical room. However, this difference was not statistically significant. Boxplot comparisons of the four samplers analysed in log_10_‐transformed units for total fungi, *Cladosporium*, and *Penicillium* species indicated that the data distribution was approximately normal. Total fungal counts obtained using the RCS Plus and SAS Super 90 samplers were comparable to one another, but both yielded significantly lower cfu/m^3^ values than those collected by the Andersen and Burkard samplers (*p* < 0.0001). The 95% confidence intervals for mean fungal concentrations confirmed this trend across the tested devices (Busse and Holgate 2008).

The Andersen sampler retrieved slightly higher numbers of *Cladosporium* propagules at most locations, except for the garage, although this difference was not statistically significant when compared with the Burkard sampler. In contrast, the Burkard sampler recovered greater numbers of *Penicillium* species at three out of five locations. With regard to *Alternaria*, the Andersen sampler consistently yielded higher counts than the Burkard at four of the five sampling sites. Both the SAS Super 90 and RCS Plus samplers consistently recorded lower concentrations of total fungi compared with the Burkard and Andersen impactors at all sampling locations (Apangu et al. 2023).

In indoor fungal bioaerosol monitoring, the Coriolis μ sampler and the Andersen cascade impactor differ fundamentally in their collection principles and analytical outputs. The Coriolis μ system uses cyclonic liquid impingement to concentrate airborne particles into a liquid buffer, making it particularly suitable for molecular analyses such as qPCR and high‐throughput sequencing, and enabling detection of both viable and non‐viable fungal material. In contrast, the Andersen impactor relies on size‐segregated inertial impaction onto agar media, allowing direct enumeration of viable spores and providing aerodynamic size distribution data relevant to respiratory exposure assessment, though it may underestimate total diversity due to culture bias. It is important to note that these methods do not measure the same biological signal: the Andersen impactor quantifies viable propagules capable of forming colonies; the Coriolis μ captures both viable and non‐viable material suitable for molecular detection; and filter‐based methods recover total fungal DNA regardless of cell viability. These differences must be considered when comparing results across methods. Thus, the Coriolis μ sampler is advantageous for comprehensive molecular characterization, whereas the Andersen method excels in viable quantification and size‐resolved exposure analysis. Volumetric spore traps commonly used in aerobiological monitoring were not included in the primary comparison, as they are predominantly applied for outdoor seasonal surveillance rather than quantitative indoor fungal exposure assessment (Al Hallak et al. 2025). A comparative summary of the principal characteristics, strengths, limitations, and recommended applications of the commonly used indoor fungal bioaerosol sampling methods is presented in Table 1.

**TABLE 1:** Comparison table of different methods for indoor fungal bioaerosol.

	Impact method	Coriolis μ method	Filter based air sampling	Sedimentation method
Practicality	Moderate	Less	More practical	More practical
Versatility	Moderate	Less	Less	Most
Molecular analysis	Yes	Yes	Yes	No
Detecting fungus species	Moderate	Moderate	Less	No
Cost	Moderate	High	Less	Very low
Volumetric	Yes	Yes	Yes	No
Culturable	Yes	Yes	Yes	Yes
Detect small particles (< 2 μm)	Moderate	High	Low	Very low
Quantitative accuracy	High	High	Moderate	Low
Taxonomic accuracy	Moderate	High	Moderate	Low
Viability accuracy	High	Moderate	Less	High
Reproducibility	High	Moderate	Moderate	Low
Bias limitations	Culture bias; possible viability loss on impact	Dilution effects; liquid loss; equipment complexity	Extraction efficiency bias; filter retention variability	Non‐volumetric; strongly influenced by airflow, gravity, and surface placement
Practical deployment	Moderate (requires calibration, electricity, trained handling)	Low–Moderate (portable but technically demanding and maintenance‐intensive)	Moderate (portable pump required; adaptable to various settings)	High (simple, low‐cost, no power required)
Best suited for	Viable concentration assessment and size‐resolved exposure analysis	Integrated molecular detection and total fungal load characterization	Combined culture‐independent and molecular diversity studies	Preliminary screening and low‐resource monitoring
References	(King et al. 2020); (Ngashangva et al. 2022); (Hardwick et al. 2017)	(Zhu et al. 2025); (Yinon 1999); (Al Hallak et al. 2025)	(King et al. 2020); (Ngashangva et al. 2022); (Lee, Low, et al. 2025; Yan et al. 2025)	(King et al. 2020); (Ngashangva et al. 2022); (Thompson et al. 1994); (Hardwick et al. 2017)

*Note:* Versatility: Suitability across different environments and compatibility with culture‐based and molecular analyses, Volumetric: Collection from a defined air volume, enabling standardized concentration units (e.g., CFU/m^3^ or gene copies/m^3^), Reproducibility: Consistency of results across repeated measurements under standardized conditions, Bias limitations: Primary source of systematic error intrinsic to the method (e.g., culture bias, liquid loss, extraction efficiency, non‐volumetric deposition), Practical deployment: Ease of field use with respect to power supply, equipment size, calibration, and operator training, Best suited for: Primary application context based on the method's combined strengths (e.g., viable quantification, molecular profiling, exposure screening), Quantitative accuracy: ability to provide volumetrically normalized fungal concentrations (e.g., CFU/m^3^ or spore equivalents/m^3^), Taxonomic accuracy: breadth and resolution of fungal species identification, Viability accuracy: ability to distinguish between viable and non‐viable fungal propagules.

Taken together, these limitations reflect a fundamental trade‐off: impaction methods preserve volumetric precision and viability information but underestimate total fungal diversity; liquid cyclone sampling enables molecular breadth but introduces liquid loss and potential reaerosolization artefacts; and filter‐based collection maximizes recovery of non‐viable and low‐abundance taxa but is sensitive to extraction efficiency and DNA inhibition. No single method simultaneously optimizes all three dimensions, which reinforces the case for integrated sampling strategies in comprehensive indoor fungal assessments. Future research should focus on developing standardized hybrid approaches that integrate size‐fractionated sampling with molecular detection, alongside inter‐laboratory validation studies to improve comparability, recovery efficiency assessment, and the establishment of harmonized fungal exposure metrics (Kabir et al. 2020).

### 3.6 Computational and Operational Data Evaluation (CODE) Frameworks

Computational and data‐driven approaches are increasingly applied to interpret fungal bioaerosol datasets generated through physical sampling and molecular analyses (e.g., CFU data from impactors, DNA extracted from filters or liquid samplers, qPCR outputs, sequencing datasets, and associated environmental metadata such as temperature and humidity). These approaches do not constitute primary sampling techniques; rather, they function as downstream analytical frameworks that integrate concentration measurements with environmental, meteorological, and building‐related parameters to enhance interpretive and predictive capacity. Data integration frameworks combine volumetric fungal concentration data with variables such as temperature, relative humidity, ventilation rate, occupancy density, and seasonal indicators. Through statistical modelling, multivariate analysis, and predictive algorithms, these approaches enable identification of temporal trends, source contributions, and environmental drivers influencing fungal aerosolization. By synthesizing heterogeneous datasets, computational analysis facilitates detection of patterns that may not be evident through conventional descriptive statistics (Choi et al. 2024; Lee, Jeong, et al. 2025; Wang et al. 2025).

The strength of such frameworks lies in their capacity to handle complex, high‐dimensional datasets, particularly when longitudinal monitoring or multi‐site comparisons are involved. Integration of time‐series analysis and regression modelling allows evaluation of seasonal dynamics and exposure variability. In advanced applications, predictive models can estimate fungal concentrations under varying environmental scenarios, supporting risk assessment and indoor air quality management strategies. However, the reliability of computational analysis is fundamentally dependent on the quality, representativeness, and standardization of upstream sampling and analytical procedures. Variability in airflow calibration, sampling duration, filter recovery efficiency, or DNA extraction protocols may introduce bias that propagates through modelling workflows. Computational modelling requires methodological expertise and adequate computational resources, particularly when high‐resolution spatial or temporal datasets are analysed (Kour et al. 2025; Wang et al. 2025).

## 4 Conclusion

Fungi present in indoor environments exhibit substantial diversity, dynamic behaviour, and varying degrees of pathogenic potential for human health. Although moisture intrusion, inadequate ventilation, and building‐related factors such as SBS are widely recognized drivers of indoor fungal proliferation, their interactions with airborne fungal dynamics remain insufficiently characterized.

Despite growing interest in indoor and outdoor microbiome research, fungal communities remain substantially less studied than bacterial counterparts, particularly with respect to long‐term temporal dynamics, functional traits, and exposure‐relevant metrics. Current research is further hindered by methodological heterogeneity, a lack of standardized sampling and analytical protocols, and inconsistent integration of culture‐based, molecular, and computational approaches. Together, these limitations reduce comparability across studies and preclude the formulation of novel, mechanistic research questions linking fungal sources, aerosolization pathways, and health outcomes. Future investigations should therefore prioritise harmonised methodologies, longitudinal study designs, and integrated analytical frameworks that bridge indoor and outdoor mycobiome research. A major unresolved challenge remains the absence of harmonised, evidence‐based standards for assessing indoor air quality using fungal spore concentrations and species composition. This limitation complicates both cross‐study comparisons and the translation of research findings into actionable guidance for indoor air quality management. Occupational environments, such as sawmills, further demonstrate how dominant airborne fungal taxa and exposure profiles vary substantially by setting, underscoring the need for context‐specific sampling and assessment strategies.

The comparison of commonly applied sampling approaches including Andersen cascade impactors, sedimentation methods, and advanced molecular and computational analytical frameworks demonstrates that no single method can comprehensively characterize airborne indoor fungal exposure. Culture‐based samplers provide valuable insights into viable fungal populations but are constrained by selective growth conditions and underestimation of total fungal diversity, while passive methods lack volumetric precision. Molecular and computational analyses enhance taxonomic resolution and interpretive power but remain dependent on the quality and representativeness of primary air sampling data. These methodological trade‐offs highlight a critical research gap: the absence of integrated, standardized strategies that combine complementary methods to simultaneously capture fungal concentration, diversity, and exposure relevance. Addressing this gap is essential for improving comparability across studies and advancing robust, evidence‐based assessment of indoor fungal bioaerosols. Measurement of airborne fungal concentrations cannot substitute for building‐level moisture control strategies. Instead, sampling methods should be integrated within broader indoor air quality management frameworks.

## Author Contributions

**Juhi Mishra:** conceptualization, methodology, writing – original draft, funding acquisition, writing – review and editing. **Wioletta Przystas:** investigation, supervision, writing – review and editing.

## Funding

This work was supported by Silesian University of Technology, 08/020/BKM25/0050.

## Disclosure

During the preparation of this work, the author (Juhi Mishra) used Openai (Chatgpt 5.5) in order to improve the language, readability, and clarity of selected passages. After using this tool, the author (Juhi Mishra) reviewed and edited the content as necessary and takes full responsibility for the content of the publication. No generative AI tool was used to produce original scientific content, analyse or interpret data, generate figures or tables, or formulate scientific conclusions.

## Conflicts of Interest

The authors declare no conflicts of interest.

## Data Availability

Data sharing not applicable to this article as no datasets were generated or analysed during the current study.
